# Selected abstracts from the 2018 Simulation Summit: Ottawa, Canada. 28-29 September 2018

**DOI:** 10.1186/s41077-018-0076-3

**Published:** 2018-09-04

## 03 Risk orientation predicts hypoxic time during difficult airway simulation

### Jake Hayward, Niresha Velmurugiah, Jonathan Duff

#### University of Alberta, Edmonton, AB, Canada

**Background**

Personality factors may explain some of the practice variation observed in medicine. The extent to which personality influences resuscitation decision-making is unknown. We use simulation to investigate the relationship between individual risk orientation and difficult airway management.

**Hypothesis**

Risk orientation in Emergency Medicine trainees predicts the time of hypoxia tolerated prior to intubation of a known difficult airway.

**Methods**

Ten Emergency Medicine residents (PGY-1, PGY-2, and CCFP-EM) from the University of Alberta participated in a standardized difficult airway simulation. There was a constant rate of oxygen desaturation necessitating eventual intubation. Following the simulation participants took part in a 10-minute open-ended interview with the study investigators and completed a risk orientation questionnaire. The time of hypoxia prior to intubation was compared with questionnaire scores. Audio interviews were transcribed and examined using a theme analysis.

**Results**

Nine participants were included in the final analysis; one did not complete the simulation as instructed. Higher risk propensity predicted longer hypoxic time prior to intubation (R = 0.72, p = 0.03). Trends showed a negative correlation between risk aversion and time of hypoxia (R = -0.41, p > 0.05). Major themes that emerged were fear regarding patient instability and the team’s ability to successfully secure an airway. These themes were emphasized more by participants that intervened at higher oxygen saturations.

**Conclusions**

We demonstrate that personality characteristics play a role in resuscitation management at an early stage of training. Our results suggest that trainees might be individually susceptible to certain types of medical error. Implications for resident training, care quality, and patient safety are discussed.

## 05 The role of the challenge point framework in surgical skill training: Interaction between learner cognitive load and performance

### Reuben N. Addison^1^, Linda Rohr^2^, Adam Dubrowski^2^

#### ^1^Louisiana State University, Baton Rouge, LA, USA; ^2^Memorial University, St. John's, NL, Canada

**Background**

The challenge point framework (CPF) has been proposed as a framework that can be used to design effective simulation training programs. Previous reports have shown how curricula, based on the CPF, have significantly improved learning. In test situations, it was observed that learners who practiced with the curricula based on the CPF consistently performed better compared to those trained under traditional methods.

**Objective**

This study examines the potential underpinning mechanisms of the CPF. We hypothesize that those who practiced on the traditional bench top simulator would have superior performance as well as record lower cognitive load compared to those who did not practice.

**Methods**

Sixteen novice trainees were randomly assigned to both experimental and control groups. Participants accessed a computer-based video instruction (CBVI) of the one-hand knot-tying. Following this, only the experimental group completed ten knot-tying trials on a simple apparatus (pre-training). Trials 1, 4, 7 and 10 were performed under a dual-task condition (i.e. focus on the knot-tying task whiles attending to a light stimulus). After a week, all participants returned to practice five trials on a more complex apparatus. Trials 1, 3, and 5 were also performed under a dual-task condition. Both simple reaction time (SRT) and subjective rating of mental effort (SRME) were assessed as a measure of cognitive load while performances were video-recorded and scored using the Global Rating Scale (GRS).

**Results**

The ANOVA results indicated main effect of group condition on GRS, (*F*_(1, 14)_ = 1095.53, *p* <.05), SRT, (*F*_(1, 14)_ = 328.84, *p* <.05) and SRME, (*F*_(1, 14)_ = 184.87, *p* <.05). The experimental group recorded higher GRS scores and lower SRT and SRME compared to the control group.

**Conclusions**

Findings suggest designing a simulation training based on the CPF, leads to superior performance and lowered cognitive load.

## 06 The stressed heart: Using simulation to assess mobile measures of heart rate variability

### Vicki LeBlanc^1^, Christopher Hicks^2^, George N. Mastoras^3^, Philip Aucoin^3^, Connor O'Rielly^3^

#### ^1^University of Ottawa Skills and Simulation Centre, Ottawa, ON, Canada; ^2^St. Michael's Hospital, Toronto, ON, Canada; ^3^University of Ottawa, Ottawa, ON, Canada

**Background**

Simulation educators are interested in how stress influences learning & performance, in order to best prepare learners for stressful clinical situations. Mobile technology for measuring this stress is increasingly accessible and affordable. This is especially true for heart rate variability (HRV), which measures sympathetic vs parasympathetic activity. However, little is known regarding the ability of various HRV measures (e.g. time-domain vs frequency-domain) to detect short-term physiological stress. In this project, we used simulation modalities to create known stressful situations, in order to assess various HRV measures for their sensitivity in detecting acute stress responses.

**Methods**

Eight (8) Emergency residents participated in 2 known stressful simulation scenarios and 4 rest periods. In each phase, we measured their *subjective stress* (State-Trait Anxiety Inventory [STAI]), *physiological arousal* (avg heart rate [HR]), *time-based HRV* (rMSSD- root mean square of successive differences; SDNN- standard deviation of NN intervals; pNN50 - proportion of NN intervals that differ > 50ms) and *frequency-based HRV* (Low Frequency/High Frequency [LF/HF] ratio).

**Results**

A multivariate ANOVA, with phase (rest, scenario) as independent variable, indicates that the STAI, HR, and time-domain HRV measures showed greater stress levels in the scenarios compared to rest phases (p values < .02, ε^2^= .17 to .44). In contrast, the frequency–based HRV measure (LF/HF ratio) was not significantly different (p= .68, ε^2^ =.01). Pearson correlation coefficients revealed that two of the time-domain HRV variables (rMSSD, pNN50) were moderately to very strongly correlated with all other measures (r= .43 to .79). The frequency-based HRV measure showed very weak to moderate correlations with the other measures (r=.21 to .55).

**Conclusions**

Time-based HRV measures detected increased physiological stress during known stressors; the frequency-based HRV measure did not. Of these, rMSSD and PNN50 showed the greatest sensitivity, and the strongest correlations with all other measures. Educators and researchers seeking to measure HRV during their simulation sessions should consider using these time-based measures. Furthermore, all variables appear to measure interrelated, but different, aspects of stress responses. Educators and researchers looking at stress should incorporate measures of different systems (e.g. subjective & physiological stress, arousal) into their designs.

## 07 Validation of a simulation-based tool for formative assessment of echocardiography skill competence in cardiology trainees

### Sherryn P. Rambihar^1^, Ara Tekian^2^, Matthew Lineberry^3^, Yoon-Soo Park^2^, Gillian Nesbitt^1^, Jeremy Edwards^1^, Ryan Brydges^1^

#### ^1^University of Toronto, Toronto, ON, Canada; ^2^University of Illinois at Chicago, Chicago, IL, USA; ^3^University of Kansas Medical Center, Kansas City, KS, USA

**Background**

New training guidelines for echocardiography competence recommend a shift from time-based metrics to assessment using simulation, direct observation and multisource evaluation. A key gap is that each cardiology program must decide its own approach to implementation, and few assessment tools exist in the published literature. We developed a simulation-based echocardiography competence assessment tool (ECAT) of basic echocardiography skills. We collected validity evidence to evaluate our claim for using the ECAT for formative assessment.

**Methods**

We included cardiology trainees (n=5 C1; n=5 C2; n=4 >C3), and assessed them on a simulator using the ECAT. We video-recorded participants’ performance, which four raters assessed with in real-time or offline. After providing participants with video footage and rater feedback, we interviewed all 14 participants and 3 raters individually. To generate implications evidence, we conducted a simple content analysis of the interview data. For scoring evidence, we computed internal consistency and inter-rater reliability metrics. For extrapolation evidence, we looked at the association between ECAT scores and participants’ expertise, diagnostic scan quality, and summative exam score.

**Results**

Our analysis revealed three themes: i) feedback stimulated change, ii) how feedback was delivered impacted participants’ perceived learning, and iii) assessment credibility influenced participants’ reception of feedback. The inter-rater reliability for the ECAT score was ICC=0.913 (95% CI 0.81-0.97). An exploratory factor analysis demonstrated a two-factor model, and the coefficient alpha for each factor was 0.96 and 0.87. ECAT scores correlated with summative exam score (r=0.66, p=0.02), were associated with judgments of the diagnostic quality of participants’ scans (p=0.007), as well as the number of echocardiograms seen (p=0.014), performed (0.012) and interpreted (0.024). ECAT score increased with participants’ level of training (p=0.01); post-hoc analysis showed C2’s did not differ from >C3’s, and both cohorts outperformed C1’s.

**Conclusions**

Trainees and raters provided validity evidence we can use to refine the ECAT process, and feedback delivery for improved formative assessment. We suggest the collective evidence is favorable, supporting use of the ECAT for simulation-based formative assessment. Our study provides a process for educators seeking to develop and evaluate instruments for formative assessment and competence-based medical education.

## 09 Fulfillment of educational objectives by healthcare providers during a simulated mass casualty event

### Ali Mulla^1^, Christopher G. Heyd^1^, Emma Bridgwater^1^, Hamish Cowan^2^, Teresa M. Chan^1^

#### ^1^McMaster University, Hamilton, ON, Canada; ^2^Hamilton Health Sciences, Burlington, ON, Canada

**Background**

Training in disaster management aims to increase healthcare provider (HCP) proficiency in infrequently used skills. Consequently, some institutions engage in occasional large-scale mass casualty incident (MCI) simulation to give learners an opportunity to manage a patient surge and engage in scarcity mitigation strategies. Moreover, as HCPs are generally ineffective at self-reflection and identifying learning needs, simulation provides a platform that may help participants both recognize and address educational gaps.

**Methods**

This study aimed to evaluate how the educational needs of HCPs are met and change with participation in large-scale MCI simulation.

**Results**

A simulated MCI was conducted in a mock emergency department (ED) half the scale of the local ED. Participants included staff nurses, respiratory therapists, paramedics and emergency medicine residents. The scenario consisted of a train collision with casualties of varying ages, backgrounds, and injury severity designed to exceed the resource capacity of the department. A debriefing session immediately followed the completion of the scenario.

Pre- and post-simulation, participants completed surveys consisting of Likert-scale and open-ended questions with factors relating to scarce resource use and participant personal learning objectives. Learning objectives prior to and after the simulation were independently categorized into themes by two authors, with overall values and proportions analysed for differences.

Thirty-five multidisciplinary HCPs participated in this exercise. Total number of learning objectives identified decreased significantly post-simulation (p<0.01). Learning objectives associated with increasing confidence, practice and experience were heavily represented pre-simulation. In contrast, HCPs listed a greater number of objectives related to resource allocation post-simulation, also reporting greater confidence with these strategies after the MCI (p<0.001).

**Conclusions**

A large-scale simulated MCI increases HCP confidence in the use of scarcity mitigation strategies while fulfilling experiential and practice-related learning objectives. Targeted simulation is a useful platform to facilitate HCP self-reflection and identification of educational needs and future learning objectives.

## 12 Closing the assessment loop in the OSCEs: A case for learning analytics that inform learning patterns

### Ilian Cruz-Panesso, Valérie Chabot

#### Université de Montréal, Montréal, QC, Canada

Although formative assessment has largely proved to be an efficient tool in higher education (Yorke, 2003), its utility in the context of the OSCEs remain an area of study, in particular when addressing how its results may impact students’ capacity to develop clinical skills and knowledge (Chisnall, Vince, Hall, & Tribe 2015). This pilot study aims to determine the sensitivity of formative OSCEs in detecting different levels of students’ global performance and the effective learning activities to support progress from one level to the other. The anonymized data from formative OSCEs of 262 medical students of second year, who solved 4 cases of similar complexity, was analyzed. Global performance was appraised by individual instructors for each case on a 4-point likert scale (1 = poor performance, 4 = exemplar performance). Four different but complementary performance measures were taken into account: Medical faculty evaluated (1) completeness and pertinence of history taken, (2) precision and argumentation of clinical diagnosis, and (3) communication and attitude skills; standardized patients assessed the (4) students’ ability to communicate and empathize. Data from the four cases was aggregated and a series of ANOVA were used to assess if there was a significant difference in performance. Significant differences were found between students in the four performance categories in particular in their ability to provide a diagnosis and the reasoning factors. Statistical comparisons between poor and exemplar performers showed small but still significant differences in history intake and significant differences in all the other performance measures. These results will help to develop theoretical foundations for establishing learning trajectories that account for different levels of students’ performance and effective learning activities to support students’ progress from one level to the other.

## 13 Time and motion study to compare High-Fidelity Clinical Simulation (HFCS) with clinical rotations: Avenues for improving the HFCS approach

### Liette St-Pierre

#### Université du Québec à Trois-Rivières, Trois-Rivières, QC, Canada

In Quebec, HFCS has been used in nursing education for a number of years and many educational institutions use it in replacement of clinical rotation days. The results obtained during the study by Hayden et al. (2014) demonstrated that replacing hours of rotation by HFCS had no negative impact on the acquisition of knowledge and competencies by students who did 50% of their clinical training in a traditional rotation setting and 50% in HFCS. However, in Quebec, the Ordre des infirmières et infirmiers does not permit this practice. The goal of the project presented is to compare the activities carried out by students during the days spent in HFCS with those carried out during the clinical rotations. A time and motion study provided a detailed description of these activities, which were observed and scored on an observation checklist, which included value-added tasks and non-value added tasks. The collection of information did not focus on the quality of the tasks performed by the students, but rather on the activities in which they were involved. Ten days were necessary for observation of the HFCS days, while 20 days were observed during the supervised rotation days, in order to obtain significantly valid data. Although the format was different during the HFCS days, the material learned and the skills to be developed in working with patients were comparable on certain points, such as the preparation and planning of the intervention. However, medication preparation as well as nurse-patient contact time are aspects to be improved during the HFCS days. The time spent on debriefing in connection with the scenarios observed in HFCS is a positive point for the students’ learning. The results obtained should guide the teachers who use HFCS and promote greater realism in the deployment of the scenarios used and the educational approach in general (briefing, simulation and debriefing) in order to ensure that students have a successful and worthwhile learning experience.

## 15 Escape game as a theatre-based simulation for teamwork skills training in undergraduate medical education

### Anthony V. Seto

#### University of Calgary, Calgary, AB, Canada

**Background**

One use of theatre-based simulation in undergraduate medical education is teaching around clinical content. However, simulation content is often medically-focused, and post-simulation debriefs may not prioritize discussion of teamwork skills, as time is limited. Furthermore, debriefing both medical and teamwork aspects of a case may add to the learner’s cognitive load.

**Objective**

This education innovation’s goal was to design an escape game as a non-clinical simulation to gamify teamwork skills training, with focus on the collaborator CanMEDS role. In the entertainment industry, escape games are activities where teams solve a series of puzzles together to ultimately escape a room.

**Methods**

A simulation theatre from the University of Calgary was transformed into an escape game. Second year medical students piloted the escape game, designed to surface teamwork competencies from the four University of Calgary Team Scheme domains (adapted from CIHC’s National Interprofessional Competency Framework and TeamSTEPPS^TM^): Leadership/Membership, Communication, Situation Monitoring, and Collaborative Decision-Making/Mutual Support. Post-game, students engaged in a debrief and written reflective exercise to critically analyze successes and challenges in demonstrating Team Scheme competencies and propose solutions to challenges. Students then documented up to 3 goals on how they would further apply teamwork competencies to their own practice and/or life.

**Results**

Through the escape game pilots, students were able to demonstrate teamwork competencies, under every Team Scheme domain, which they will need to apply when collaborating with teams in future simulations and clinical practice. Students’ self-identified strengths include “identifying roles”, “call-outs”, “sharing mental models”, and “task-assistance/feedback”. Challenges include “becoming task-focused”, “not closing communication loops”, and “not reassessing”.

**Conclusions**

A teamwork simulation, in the form of an escape game, enables medical students to apply and demonstrate teamwork skills, identify strengths and challenges in their teamwork skills, and discuss areas of teamwork skills needing improvement. Advantages of this innovation include its use as an acclimatizer to the simulation training environment and process, portability and low start-up cost, transferability to non-medical disciplines, and customizability of puzzles to target specific objectives. The escape game will launch in Intro to Clinical Practice, a second-year medical student course at the University of Calgary.

## 16 Does spaced instructional design result in improved long-term retention of pediatric resuscitation skills? A randomized education study

### Catherine Patocka^1^, Adam Cheng^2^, Matthew Sibbald^3^, Tasnima Abedin^1^, Jonathan Duff^4^, Anita Lai^5^, Patricia A. Lee^1^, Helen Levin^6^, Terry Varshney^7^, Bryan Weber^1^, Farhan Bhanji^8^

#### ^1^University of Calgary, Calgary, AB, Canada; ^2^Alberta Children's Hospital, Calgary, AB, Canada; ^3^McMaster University, Oakville, ON, Canada; ^4^University of Alberta, Edmonton, AB, Canada; ^5^University of Ottawa Skills and Simulation Centre, Ottawa, ON, Canada; ^6^University of Western Ontario, London, ON, Canada; ^7^Childern’s Hospital of Eastern Ontario, Ottawa, ON, Canada; ^8^Montréal Children's Hospital, Montréal, QC, Canada

**Background**

Resuscitation performance directly impacts patient survival therefore, healthcare providers (HCP) are routinely taught these critical skills in a formal course at a significant cost to the healthcare system. HCPs completing resuscitation courses routinely show a significant decay of skills in the weeks to months following training, often before they would be called upon to use them in a clinical setting.

Objective: The purpose of this study was to determine if a pediatric resuscitation course taught to experienced Emergency Medical Services providers (EMS) in a spaced format compared to the usual massed instruction results in improved retention of learner performance in a simulated pediatric resuscitation scenario.

**Methods**

We delivered a Pediatric Advanced Life Support (PALS) course to EMS providers in either a spaced (four 3.5-hour weekly sessions) or a massed format which reflects conventional resuscitation course delivery (two 7-hour sessions over 2 consecutive days). Before and 12-weeks following course completion, participants completed a simulated pediatric resuscitation scenario in teams of 2 providers. Blinded observers used the clinical performance tool (CPT), a resuscitation scenario assessment tool with prior published evidence of validity, to evaluate video recordings of participant performance (primary outcome). Time to cardiopulmonary resuscitation (CPR) and chest compression fraction (% time during which chest compressions were done) were also recorded as secondary outcomes.

**Results**

16 out of 22 teams (7 spaced and 9 massed) of providers completed the baseline and post-12 weeks resuscitation scenarios. There was no significant difference between the spaced and massed groups on the baseline CPT score (40 ±4.6 versus 35 ±4.1, *p*=0.51). Teams in the spaced group performed significantly better (57 ±4.3 versus 40 ±3.8, *p*=0.0001) on the 12-weeks post-course assessment. There was no difference between time to CPR at baseline (59 ±15s versus 66 ±13s, *p*=0.96) or at the 12-week post-course assessment (32 ±5.2s versus 23 ±4.6s, *p*=0.18) and chest compression fraction baseline (76 ± 4.7% versus 70 ±4.1%, *p*=0.36) 12-weeks post-course (87± 3.8% versus 81 ±3.3%, *p*=0.45).

**Conclusions**

Long-term retention of pediatric resuscitation performance is better three-months post-training when it is learned in a spaced format compared to traditional massed training.

